# Solid-State Green Synthesis of Ag NPs: Higher Temperature Harvests Larger Ag NPs but Smaller Size Has Better Catalytic Reduction Reaction

**DOI:** 10.1038/s41598-019-51693-w

**Published:** 2019-10-23

**Authors:** Dina Sadeq Al-Namil, Elsy El Khoury, Digambara Patra

**Affiliations:** Department of Chemistry, American University of Beirut, Beirut, Lebanon

**Keywords:** Chemistry, Green chemistry, Materials chemistry, Physical chemistry, Process chemistry

## Abstract

In this work a novel solid-state green approach without using any solvent environment has been proposed to synthesize Ag NPs. The synthetic condition has been investigated in 4 °C, 20 °C, 40 °C and 60 °C and at ten different time intervals. This synthesis process gives different size and shape of curcumin conjugated Ag NPs, which have been confirmed by various morphological and spectroscopic techniques. It is found that higher temperature and longer time produces larger particles size and different varieties in shapes. For example, Ag NPs prepared at 4 °C are spherical shapes whereas that prepared at 60 °C are of spherical, rods, and many hexagonal shapes. At 60 °C and after 5 and 7 days the size of the prepared Ag NPs exceed the nano scale to reach micro scale level. This size and shape distribution are well reflected in the optical properties as absorbance, fluorescence intensity and SFS intensity of Ag NPs consistently increase with increase in temperature during synthesis. Ag NPs obtained in different temperature and various time intervals have been subsequently tested as catalysts for the reduction reaction, where 4-nitrophenol is reduced to 4-aminophenol in the presence of NaBH_4_. It is found that smaller particles have better catalytic properties for the reduction reaction.

## Introduction

Silver nanoparticles (Ag NPs) of different shapes and sizes can be prepared by controlling the synthetic process^1–10^. Normally organic/inorganic materials are often prepared in a solvent environment, however, in solid state synthesis, materials are prepared in solid state by mixing/grinding. In solid state synthesis procedure amount of solvent used is limited and it also helps in making distinctive morphologies as well as compositions that may be useful in advanced materials such as piezoelectrics, scintillation crystals, etc.^11^. Thus, to make materials of required functionalities, this solid-state synthesis approach is a green method^12^. To control the size and shape of the matter, solid state methods can have more advantages to prepare nanoparticles and tune their properties^11,12^.

Synthesis of silver nanoparticles is an area that has witnessed much enthusiasm in recent time^13^. These Ag nanoparticles have exceptional optical, electrical, chemical and magnetic properties which are not same as that obtained for their bulk match. Crystalline structure, composition, size, and shape of Ag NPs decide their properties. Ag NPs are applied in extensive range of potential used in various areas, which include catalysis, medicine and biotechnology and catalysis due to those exclusive properties. Colloidal nanoparticles of Ag is among the most widely investigated nanoscale materials^14^. However, it can be achieved to tune the properties of Ag NPs in the desired size and shape using the parameters discussed above^15,16^.

The main applications of silver nanoparticles are found in catalysis and as bactericides. For instance, Acacia Nilotica pod mediated Ag NPs modified glassy carbon electrode has been reported to have greater catalytic activity when reducing benzyl chloride molecule compared to the use of glassy carbon and metallic Ag electrode^17^. The synthesized Ag NPs using the extract of Gloriosa superba is based on the electron relay phenomena which will induce the degradation of methylene blue at the end of the 30 min^17^. In this manuscript, we report for the synthesis of Ag NPs using curcumin via solid state green synthetic approach. Curcumin, considered as non-toxic and safe reagent, was found to be an excellent candidate to use in the preparation of nanoparticles through green synthesis mechanism. Synergetic reducing technique has been optimized in our laboratory for the synthesis of silver nano-particles using the curcumin as reducing agent at room temperature (~20 °C), 4 °C, 40 °C and 60 °C respectively. The temperature can have an effect on the size and the shape of silver nanoparticles. The role of temperature in the formation and growth of silver nanoparticles has been identified by scanning electron microscopy (SEM) and UV–VIS spectrometer techniques. Finally, the prepared curcumin conjugated Ag NPs have been tested as catalyst for the reduction reaction of p-nitrophenol.

## Materials and Methods

### Materials

Most of the chemicals p-nitro phenol, silver nitrate, curcumin, sodium borohydride (NaBH_4_) and ethanol were all obtained from Sigma–Aldrich and directly used without further purification. For catalysis 3.4 mg of NaBH_4_ in 3 mL double distilled water (DDW) was prepared freshly.

### Preparation of Ag NPs using curcumin

For the preparation, a slightly modified method that we have reported earlier^18^ was used. Briefly, using a pestle curcumin (184.19 mg, ~0.5 mol) and silver nitrate (169.7 mg, ~1.0 mol) were together grinded in a marble mortar in the solid phase, grinding was continued until all the crystals crushed, thus, turned into powder. The color changed from yellow to dark orange (depending on the temperature it took from 30 min to 60 min) while grinding. 100 mL ethanol was then added to the powder mixture. Two each 50 mL tubes were used where the solution was transferred. The total volume of the solution was approximately 95 mL. Subsequently, growth of Ag NPs continued in ethanol for 1, 2, 4, 8, and 16 hours and for 1, 2, 3, 5, and 7 days at four different temperatures (at 4 °C, 20 °C, 40 °C, and 60 °C). Afterwards the solution was incubated for the specific time mentioned above in the tubes and solutions were centrifuged at 4000 rpm for 20 minutes at 20 °C. In order to eliminate all the unreacted and unbounded curcumin present in the solutions, ethanol wash was carried out and centrifugation was done for several time until getting a clear supernatant. Finally, 10 mL of DDW were added to the precipitate and stored the final solution. For characterization using various techniques such as, UV-visible absorption spectrophotometer, fluorometer, SEM etc., the stored sample was diluted 3 times (1 mL diluted to 3 mL of water).

### Instrumentation

Using a JASCO V-570 UV–VIS–NIR spectrophotometer, the UV-visible absorption spectra were measured at room temperature. A Bruker d8 discover X-Ray diffractometer equipped with Cu-Kα radiation (λ = 1.5405 Å) was used to collect X-Ray diffraction (XRD) data. In this case the monochromater was Johansson Type. Jobin-Yvon-Horiba Fluor log III fluorimeter (with a 100 W Xenon lamp as excitation source and R-928 operating at a voltage of 950 V as detector) was applied to measure emission and excitation fluorescence spectra where the resolution increment was 1 nm and slit was kept 5 nm. Data were analyzed using Fluorescence program. For measuring resonance Rayleigh scattering, synchronous fluorescence scan was applied in the same instrument but in this mode the excitation and emission wavelength interval (Δλ) was kept at 0 nm. Using a Netzsch TGA 209 in the temperature range 30 °C to 1000 °C with an increment of 10 K/min in a N_2_ atmosphere, all the thermogravimetric Analysis (TGA) was carried out. SEM analysis was made using Tuscan. Ultracentrifugation technique was used for separation and washing the samples.

## Results and Discussion

To understand the solid state synthesis of Ag NPs, a control was prepared by grinding 1 mmol of silver nitrate in a mortar using a pestle, until the powder was very fine. The salt was then dissolved in ethanol and sonicated and put in 2 tubes (27.5 mL each). After two days the tubes were put in a centrifuge at 4000 rpm at 25 °C for 20 minutes (control). No residue was obtained as seen in Figure S1A (see Supporting Information, SI). In the second case (sample 2), 1 mmol of silver nitrate was grounded in a mortar using a pestle, when the powder was very fine, 0.05 mmol of curcumin was added and the reagents were grounded together at room temperature until the color changed from the initially yellow to dark orange (for about 10 minutes) as depicted on Figure S2 (see SI). This may be a result of association of Ag^+^ with curcumin and could be an indication of reduction process. The mixture was washed and dissolved in ethanol then immediately centrifuged. The obtained residue (see Figure S1C, SI) was analyzed subsequently. In the third case (sample 1), the sample obtained in second case was put in 2 tubes (40 mL each). After two days (the sample was kept in ethanol for 2 days to see growth of Ag NPs in ethanol medium), the solutions were centrifuged at 4000 rpm at 25 °C for 20 minutes and the residue was collected (see Figure S1B, SI) and resuspended in ethanol by sonication and centrifuged again under the same conditions. Initially, all these three samples were analyzed by UV-visible absorption spectroscopy. As can be seen in Figure S3, the control did not show any absorption for Ag NP indicating that immediate ethanol wash does not reduce the silver ions. In the second case for solid state synthesis, Ag NPs were obtained but the yield was small (see Figure S1C, SI) and showed absorption for Ag NPs (see Figure S3). In the third case when the solution was incubated at room temperature for two days, the yield was better and showed absorption for Ag NPs with higher absorbance. Solid state synthesis of Ag NPs in the presence of curcumin (sample 2) was also confirmed by SEM and EDX analysis as given in Figure S4.

**Figure 1:**
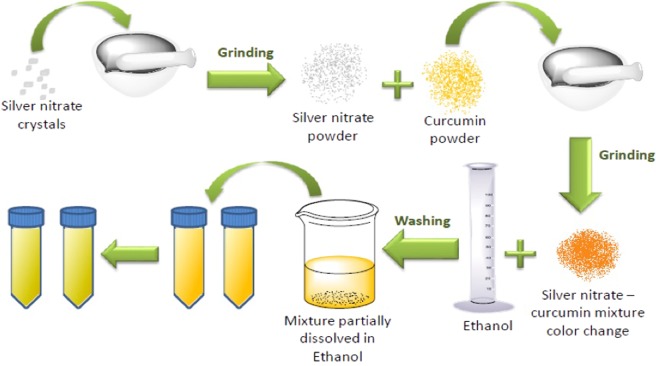
Preparation of curcumin mediated Ag NPs prepared by green solid state procedure.

Subsequently, the curcumin conjugate Ag NPs were prepared in the solid state process using silver nitrate and curcumin reacting as reducing agent, where Ag^+^ ions were reduced to metallic form Ag^0^ and particles were grown in ethanol at four different temperatures 4 °C, 20 °C, 40 °C and 60 °C as shown in Fig. 1. The two reagents were mixed and grinded till the color turn from yellow to orange. Then the solid mixture was then dispersed in ethanol and kept for 1 day for further growth. Ethanol medium was also helpful to prevent O_2_ from entering the mixture and forming silver oxide. To collect Ag NPs, for each time period/interval, the sample was centrifuged for 20 minutes and washed it by ethanol until all the unreacted curcumin was removed from the Ag NPs surfaces, this was confirmed when the washed out ethanol turned transparent instead of yellow color (due to unreacted curcumin). After having the transparent solution, double distilled water was added to Ag NPs precipitated and the solution was stored for further characterization and analysis.

**Figure 2:**
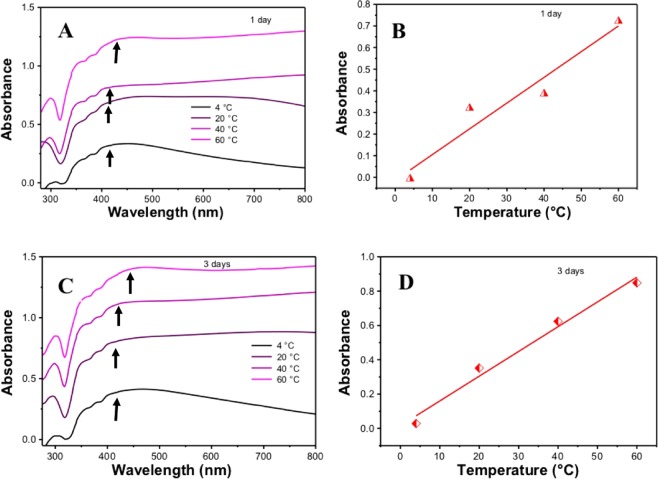
UV-visible absorption spectra of curcumin mediated Ag NPs prepared by green solid state procedure at different temperatures after 1 day (**A**) and 3 days (**C**). Variation of absorbance with temperature during preparation of curcumin mediated Ag NPs prepared by green solid state procedure after 1 day (**C**) and 3 days (**D**).

The UV-visible spectra obtained for the four different temperatures are given in Fig. 2A,C. Curcumin has two major absorption peak, one at ~266 nm for S_1_–S_2_ transition and other at ~425 nm for S_0_–S_1_ transition in aqueous medium^19^. However, the Ag NPs absorbance peaks were found around 400–450 nm in all the four temperatures. The absorbance peaks for Ag NPs appear due to the elastic scattering phenomenon as a result of Surface Plasmon resonance (SPR). Sharp peaks were noticed in UV region for 4 °C and 20 °C whereas for 40 °C and 60 °C it shifted to a higher wavelength, this is due to Ag^+^ bounded to curcumin^20^.

When we did a comparison by keeping the time fixed (1 day or 3 days) and changing the temperature only, as shown in Fig. 2A,C, it was observed that at higher temperature, the peak shifted to higher wavelength than 40 °C, 20 °C and 4 °C. This happened because with higher temperature larger size particles are formed, which shift the absorption to the longer wavelength. Curcumin has the ability to chelate on the cation metals like Zn^+2^ and Cu^+2^ (M-L) whereby those cations bind to C = O group of the ß-diketone moiety on curcumin^21^. Interestingly, temperature during preparation of Ag NPs had an impact on the absorbance of prepared Ag NPs after 1 day (see Fig. 2B,D). The absorbance increased with increase in the temperature during preparation.

**Figure 3:**
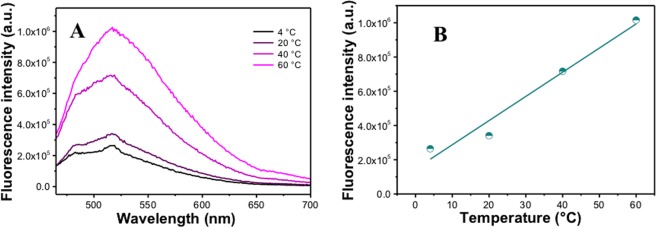
(**A**) Fluorescence spectra of curcumin mediated Ag NPs prepared by green solid state procedure at different temperatures after 1 day; (**B**) Variation of fluorescence intensity with temperature during preparation of curcumin mediated Ag NPs prepared by green solid state procedure after 1 day.

The fluorescence spectra of these Ag NPs were measured at excitation wavelength λ = 425 nm and depicted in Fig. 3A. The fluorescence showed a broad spectrum with a maximum at ~515 nm, which is similar to fluorescence spectrum of curcumin. Normally curcumin in DDW has a maximum around ~540 nm, thus this shift is an indication of conjugated curcumin at the surface of Ag NPs. Similar to the absorbance, the fluorescence intensity increased with temperature during preparation of Ag NPs as presented in Fig. 3B. The difference in the absorbance peaks and fluorescence intensity is essentially affected by the sizes and shapes of Ag NPs obtained. The Resonance Rayleigh Scattering (RRS) spectra as shown in Figure S5A was measured by applying synchronous fluorescence scan method by keeping the wavelength interval (∆λ) at 0 nm. The Resonance Rayleigh Scattering spectrum of curcumin conjugated Ag NPs at four different temperatures with ten different growth times showed four bands. The first two bands are the due to excitation bands which is similar to what we have in UV-VIS spectra in Figure S5A, and the other two bands are around emission region of curcumin conjugated Ag NPs particles. As expected the SFS intensity is highly affected by the size, where bigger particles have higher SFS or RRS intensity. Also when the temperature increased, the SFS intensity increased from ~1.3 × 10^8^ in 4 °C till ~4.6 × 10^8^ in 60 °C at wavelength maximum as shown in Figure S5B.

**Figure 4:**
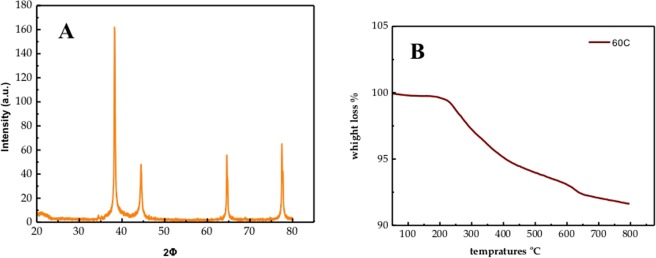
(**A**) XRD pattern of curcumin conjugated Ag NPs prepared after 1day at 60 °C; (**B**) TGA of the curcumin conjugated Ag NPs sample prepared at 60 °C and grown for 1 day.

To further confirm formation Ag NPs, the particles were collected by using the freeze drier to evaporate all the water that we used to store the samples with. The solid samples were used to measure XRD. The XRD patterns of curcumin conjugated Ag NPs for sample 2 (grown in ethanol for 1 day at 60 °C) and sample 1 (without ethanol, taken immediately after grinding and washing) is shown in Fig. 4A and Figure S6, respectively. Both of them showed similar pattern and confirmed formation of Ag NPs. The sharp Bragg reflection in the XRD indicates the association of organic molecule to the Ag NPs. All the major peaks in XRD can be indexed for the face centered cubic structure revealing the crystallinity property of the curcumin conjugated Ag NPs. The different peaks originated from the (1 1 1), (2 0 0), (2 2 0), and (3 1 1) planes of Ag NP, which perfectly match with the JCPDS card number 4-0783^22^. A major peak at lower 2 *θ* value might be due to the organic content of curcumin. The average diameter of silver nanoparticles can be estimated from the (111) diffraction peak using Scherrer’s equation as L = 0.91 λ/(ß cos α)^23^ where L is the mean crystallite size, λ the wavelength of incident rays (1.5405 Å), ß is the full width at half maximum (FWHM) of the highest intensity peak in radians, and α is the center angle of the peak in radian. The mean crystallite range for Ag NPs was determined to be 8.47 nm using this formula. This is lower than the values found in the SEM images, which will be discussed later on. Thermogravimetric analysis is presented in Fig. 4B, for the Ag NPs obtained at 60 °C within the one day sample. The decomposition temperature for curcumin from literature is approximately at 400 °C and with a minor contribution at 200 °C^24^, so it has a wide range of decomposition temperatures. In Fig. 4B it is found that a wide range of decomposition temperature that started at approximately 250 °C till 600 °C, which is similar to decomposition temperatures of curcumin. In this range, the weight loss mass of the organic compound was approximately ~8%. This indicates the presence of only silver nanoparticles in the sample, and that all the AgNO_3_ reacted with curcumin and formed Ag NPs and unreacted AgNO_3_ were completely washed out.

**Figure 5:**
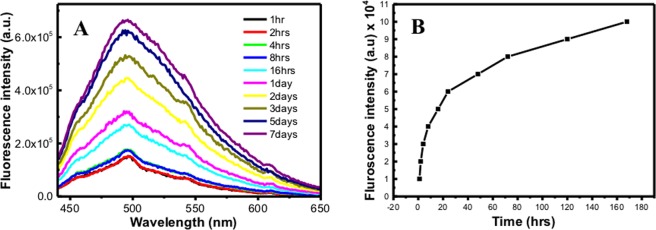
(**A**) Fluorescence emission spectra of curcumin conjugated Ag NPs in different growth time intervals at 425 nm excitation wavelength at 60 °C; (**B**) Fluorescence emission intensity at the maximum vs growth time in hours for curcumin conjugated Ag NPs at 425 nm excitation wavelength at 60 °C.

To understand the kinetics, the solid mixture was dispersed in ethanol and growth of Ag NPs was monitored at ten different times 1, 2, 4, 8 and 16 hours and after 1, 2, 3, 5, and 7 days. We noticed different peaks and intensities in the UV-visible spectra for the different growth time samples due to the different sizes and shapes. With the 1, 2, 3, 5, 7 days growth samples, the peak position shifted to higher wavelength than the 1, 2, 4, 8, 16 hours growth samples, which is due to bigger size of the particles at longer growth time. Similarly, the fluorescence for the different growth time samples as given in Fig. 5A showed broad spectra without any remarkable change in fluorescence maximum. But the fluorescence intensity of Ag NPs consistently increased with increase in growth time as provided in Fig. 5B, which suggests growth of Ag NPs increase higher conjugation of curcumin around the nanoparticles.

**Figure 6:**
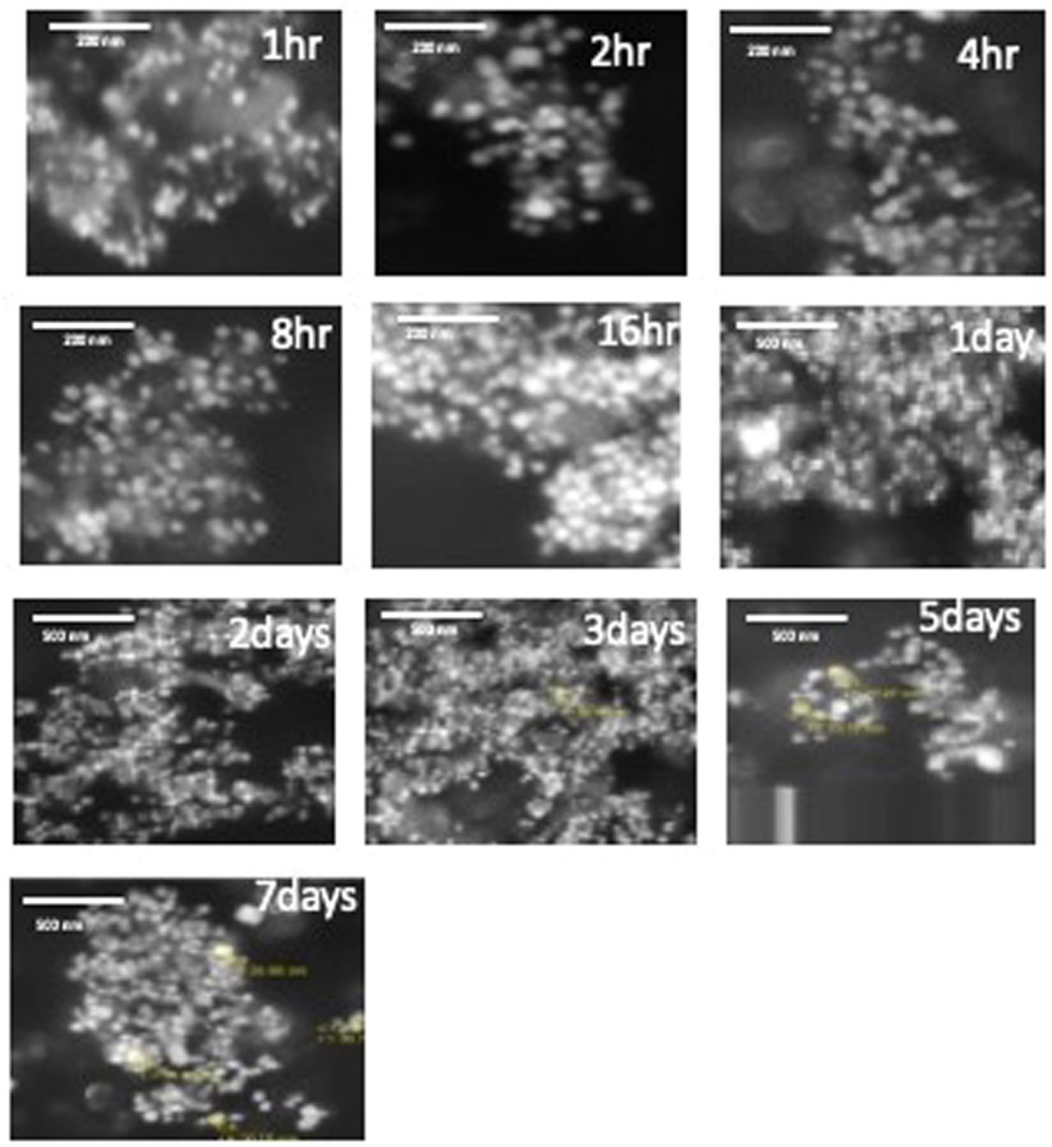
SEM images of curcumin conjugated Ag NPs in different growth time intervals at 4 °C.

**Figure 7:**
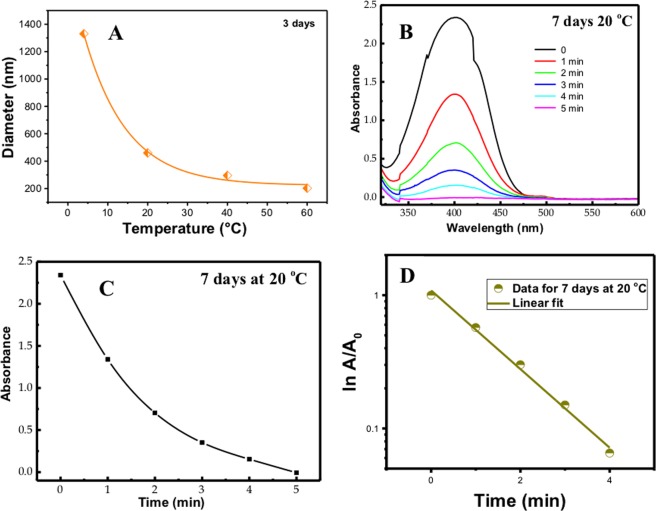
(**A**) Diameter of the Ag NPs prepared at different temperature as obtained from DLS measurement; (**B**) Change in the absorbance of 4-nitrophenol at ~400 nm in the presence of NaBH_4_ with curcumin conjugated Ag NPs prepared at 20 °C; (**C**) Absorbance change at 400 nm of 4-nitrophenol in the presence of NaBH_4_ during curcumin conjugated Ag NPs prepared at 20 °C; (**D**) Change in ln (A/A_o_) with time during reduction of 4-nitrophenol in the presence of NaBH_4_ with curcumin conjugated Ag NPs prepared at 20 °C.

The SEM images showed variation in the size of Ag NPs for the ten different times in all the four different temperatures under studied. Figure 6 at 4 °C shows how the particles became bigger with increase in growth time at the same temperatures. The 1-hour growth sample showed a very small particle size for which we could not measure the radius, but from one day till seven days growth samples the particles size diameter ranged between 15 and 40 nm, and there were particles having smaller size with mostly a spherical shape. At 20 °C shown in Figure S7 the particles became bigger with more variety in shapes like spherical and hexagonal shapes. The particles size of the 1-hour growth sample are very small in such a way that we could not measure the particles diameter. From one day till seven days samples, the size ranged between 40 and 90 nm, in this case the particles shapes were spherical, hexagonal and we noticed formation of rod-shaped particles. At 40 °C, given in Figure S8, the size of the particles ranged between 20 and 100 nm. More variety in particles shapes spherical, hexagonal and rods shapes were found. Figure S9 presents the ten samples at 60 °C, in this case the particles size were bigger, and the size ranged from 30 to 130 nm, the shapes are mostly hexagonal with spheres and rods shapes. At all four temperatures under investigation, we could see a good amount of particle aggregation. With different sizes and shapes in each sample, also many of the SEM pictures showed imperfection, some blurriness or unclear images which may be due to the presence of unreacted curcumin in the sample. During the sample preparation, we noticed there was a change in sample color from light yellow in the one-hour growth sample to dark yellow or orange in the seven days growth sample. Ag NPs prepared at 60 °C gave bigger particles than 40 °C. 20 °C gave bigger particles than 4 °C. To enlarge the particles or form cylinders, triangles or hexagons shapes particles during incubation at different temperature, the extra silver is coming from the unreacted Ag^+^ ion present in the solution. However, DLS data as depicted in Fig. 7A shows that increase in the temperature during synthesis decreases the particles size, which confirms aggregation of small Ag NPs prepared at lower temperature.

**Figure 8:**
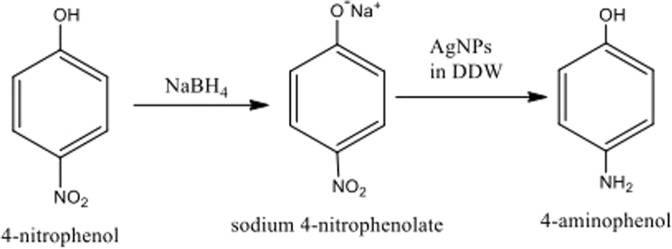
Reduction reaction of 4-nitrophenol to 4-aminophenol after the addition of NaBH_4_.

One of the important applications of silver nanoparticles is that they are used as nano-catalysts for the reduction of 4-nitrophenol with NaBH_4_ (nitro-reduction). 500 microliters (0.5 ml) of curcumin conjugated Ag NPs was added to 15 mM of NaBH_4_ that was prepared instantly, then they were mixed together with 0.15 mM of 4-nitrophenol, the total volume was equal to 3 mL. The initial color of the mixture was yellow, then it turned to transparent solution because of the reduction 4-nitrophenol to 4-aminophenol, after the addition of NaBH_4_. The reduction reaction is given in Fig. 8.

The reduction of 4-nitrophenol was observed by measuring the change in the absorbance of 4-nitrophenol at 400 nm, and to confirm that this reduction happens a peak at 290 nm was also identified for the 4-aminophenol^25,26^. To study the effect of the curcumin conjugated Ag NPs as a catalyst on the reduction of 4-nitrophenol to 4-aminophenol, first we measured the absorbance of the reduction of 4-nitrophenol by using UV-VIS spectra without curcumin conjugated Ag NPs, and then we measured the reduction reaction in the presence of curcumin conjugated Ag NPs. In the first reaction, the absorbance of 4-nitrophenol at 400 nm decreased with time, and for complete reduction we need approximately 300 hours^25^. In the second reaction absorbance of 4-nitrophenol at 400 nm was monitored in the presence of curcumin conjugated Ag NPs with time, it was found that in the presence of curcumin conjugated Ag NPs 4-nitrophenol reduced within a few minutes (12 minutes maximum) as shown in Fig. 7B,C. This is because curcumin conjugated Ag NPs will adsorb sodium borohydride and make the reduction of 4-nitrophenol to 4-aminophenol faster. Therefore, curcumin conjugated Ag NPs prepared at the different temperatures at ten different growth times can be excellent catalysts for the reduction of 4-nitrophenol.

However, different size and shape of Ag NPs were obtained at different temperatures, 20 °C, 40 °C, 60 °C, and for ten different growth times, therefore, the catalytic activities were compared for Ag NPs that were prepared in different temperatures at ten different growth times. The catalysis reaction considered to be pseudo first rate reaction because of the decreasing in intensity of the absorption peak at 400 nm over time, so a linear relation was observed between ln (A/A_o_) vs. time (see Fig. 7D), but the concentration of sodium borohydride considered to be constant. The equation below represents the linear relationship between ln (A/A_o_) and time:

$$\mathrm{ln}\,({\rm{A}}/{{\rm{A}}}_{{\rm{o}}})=-\,{\rm{\kappa }}{\rm{t}}$$

where A_o_ is the initial absorbance of the reaction system, A is the absorbance at time t, and κ is the rate constant of the chemical reduction. From this kinetic curve, the rate constant κ (s^−1^) was calculated and it shows that 7 days growth time at 60 °C has lower κ which is equal to 0.035 s^−1^. For further investigation we found that curcumin conjugated Ag NPs prepared in 20 °C served as excellent catalysts for the reduction of 4-nitrophenol based on half life period. The half-life period was calculated as below:

$${{\rm{t}}}_{{\rm{1}}/{\rm{2}}}={\rm{0.693}}/{\rm{\kappa }}$$

**Figure 9:**
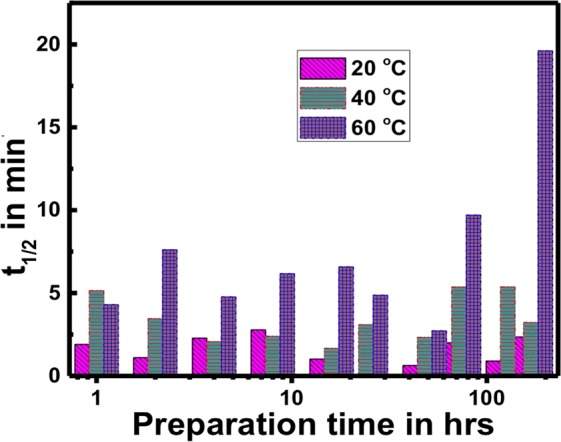
Change in t_1/2_ with the growth (preparation) times at four different temperatures, 20 °C, 40 °C and 60 °C.

It shows that 20 °C have the lowest t_1/2_ and 60 °C have the highest t_1/2_ as depicted in Fig. 9. This enhancement must be due to the different shape and size of Ag NPs in the different temperatures and growth times like we observed in the SEM images depicted in Fig. 6, S7–S9, and from those Figures we can see that samples prepared at 20 °C have in general smaller particles compared to that prepared at 40 °C and 60 °C, smaller size also suggests higher surface to volume ratio of the particles. From different reviews of the literature it has been agreed that the reduction of 4-nitrophenol to 4-aminophenol in the presence of NaBH_4_ was held on the surface of the Ag NPs, and that Ag NPs adsorb the H_2_, the hydrogen (H_2_) is released when NaBH_4_ reduces water to hydrogen like the equation:

$${{\rm{NaBH}}}_{{\rm{4}}}+{{\rm{2H}}}_{{\rm{2}}}{\rm{O}}\to {{\rm{NaBO}}}_{{\rm{2}}}+{{\rm{4H}}}_{{\rm{2}}}$$

**Figure 10:**
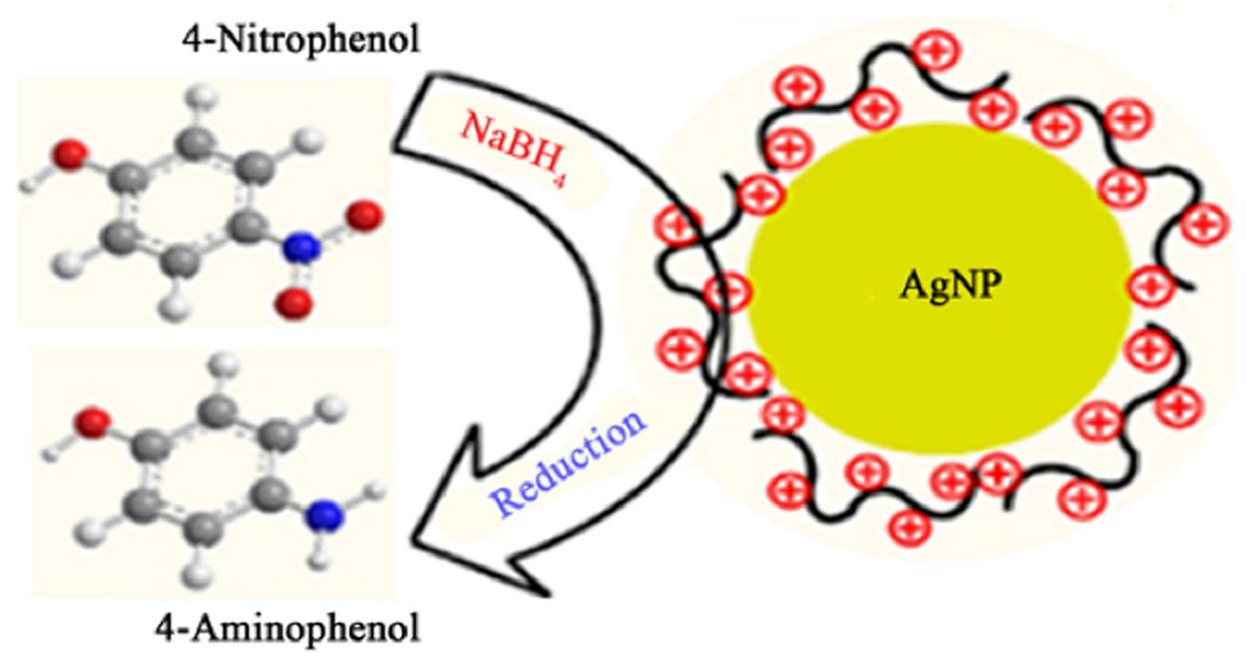
The mechanism of Ag NPs as catalyst during reduction reaction for 4-NP.

Curcumin conjugated Ag NPs act as a hydrogen carrier and the H will be transported from NaBH_4_ to 4-NP. Then the molecules of 4-NP lose electrons, to form 4-aminophenol then the latter will desorb from the surface of curcumin conjugated Ag NPs surface as shown in Fig. 10^26^.

## Conclusion

Synthesis of curcumin conjugated Ag NPs was reported using a solid-state green approach without using any solvent environment. Ag NPs synthesized in four different temperatures 4 °C, 20 °C, 40 °C and 60 °C and at ten different time intervals provided different size and shape of curcumin conjugated Ag NPs, as confirmed by various morphological and spectroscopic techniques. It was established that higher temperature and longer time creates larger particles size and different varieties in shapes. For example, Ag NPs prepared at 4 °C were spherical shapes and that prepared at 60 °C were of spherical, rods, and many hexagonal shapes. At 60 °C and after 5 and 7 days the size of the prepared Ag NPs reached micro scale level. This size and shape distribution were also reflected in the optical properties. It was noitice that absorbance, fluorescence intensity and SFS intensity of Ag NPs consistently increased with increase in temperature during synthesis. Ag NPs obtained in different temperature and various time intervals were subsequently tested as catalysts for the reduction reaction of 4-nitrophenol to 4-aminophenol using NaBH_4_ as a reducing reagent. It was shown that smaller particles have better catalytic properties for the reduction reaction, thus, using solid state green synthesis approach size and shape of nanoparticles can be tuned for its better applications such as in catalysis.

## Supplementary information

Supplemental Material

## References

[1] Abid, J. P., Wark, A. W., Brevet, P. F. & Girault, H. H. Preparation of silver nanoparticles in solution from a silver salt by laser irradiation. *Chem. Commun*. 792–793 (2002). 10.1039/b200272h 12119726

[2] Itakura T, Torigoe K, Esumi K (1995). Preparation and characterization of ultrafine metal particles in ethanol by UV irradiation using a photoinitiator. Langmuir.

[3] Pol VG (2002). Sonochemical deposition of silver nanoparticles on silica spheres. Langmuir.

[4] Stiger RM, Gorer S, Craft B, Penner PM (1999). Investigations of electrochemical silver nanocrystal growth on hydrogen-terminated silicon (100). Langmuir.

[5] Harfenist SA, Wang ZL, Alvarez MM, Vezmar I, Whetten RL (1996). Highly oriented molecular Ag nanocrystal arrays. J. Phys. Chem..

[6] Komarneni S, Newalkar D, Li B, Katsuki H, Bhalla AS (2002). Microwave−Polyol process for Pt and Ag nanoparticles. Langmuir.

[7] Liz-Marzán LM, Lado-Touriňo I (1996). Reduction and stabilization of silver nanoparticles in ethanol by nonionic surfactants. Langmuir.

[8] Petit C, Lixon P, Pileni MP (1993). *In situ* synthesis of silver nanocluster in AOT reverse micelles. J. Phys Chem..

[9] Heath JR, Knobler CM, Leff DV (1997). Pressure/Temperature phase diagrams and superlattices of organically functionalized metal nanocrystal monolayers:  The influence of particle size, size distribution, and surface passivant. J. Phys Chem. B.

[10] Santos IP, Liz-Marzán LM (2002). Formation of PVP-protected metal nanoparticles in DMF. Langmuir.

[11] Fahlman, B. D. What is Materials Chemistry? *In Materials chemistry*, 1–12 (Springer: 2011).

[12] Iravani S (2011). Green synthesis of metal nanoparticles using plants. Green Chem..

[13] Roy N, Barik A (2010). Green synthesis of silver nanoparticles from unexploited weed resources. Inter. J. Nanotechnol..

[14] Kim J-S (2007). Reduction of silver nitrate in ethanol by poly (N-vinylpyrrolidone). J. Indust. Engineer. Chem..

[15] Polte J (2015). Fundamental growth principles of colloidal metal nanoparticles–a new perspective. CrystEngComm.

[16] El Khoury E, Abiad M, Kassaify ZG, Patra D (2015). Green synthesis of curcumin conjugated nanosilver for the applications in nucleic acid sensing and anti-bacterial activity. Colloid. Surf. B: Biointerfac..

[17] Nurani, S. J., Saha, C. K. & Arifur Rahman Khan, M. *Silver Nanoparticles synthesis, properties, applications and future perspectives: a short review*. **10**, 117–126 (2015).

[18] Al-Namil DS, Patra D (2019). Green solid-state based curcumin mediated rhamnolipids stabilized silver nanoparticles: interaction of silver nanoparticles with cystine and albumins towards fluorescence sensing. Colloid. Surf. B: Biointerfac..

[19] El Khoury ED, Patra D (2013). Ionic liquid expedites partition of curcumin into solid gel phase but discourages partition into liquid crystalline phase of 1, 2-dimyristoyl-sn-glycero-3-phosphocholine liposomes. J. Phys. Chem. B.

[20] Kundu S, Nithiyanantham U (2013). *In situ* formation of curcumin stabilized shape-selective Ag nanostructures in aqueous solution and their pronounced SERS activity. RSC Adv..

[21] Zhao X-Z (2010). Interaction of curcumin with Zn (II) and Cu (II) ions based on experiment and theoretical calculation. J. Molecul. Struct..

[22] Park H-H, Zhang X, Choi Y-J, Park H-H, Hill RH (2011). Synthesis of Ag nanostructures by photochemical reduction using citrate-capped Pt seeds. J. Nanomater..

[23] Sheldon, R. A., Arends, I. W. & Hanefeld, U. Introduction: Green chemistry and catalysis. Green Chem. *Catalysis* 1–47 (2007).

[24] Kumar SSD, Mahesh A, Mahadevan S, Baran Mandal A (2014). Synthesis and characterization of curcumin loaded polymer/lipid based nanoparticles and evaluation of their antitumor effects on MCF-7 cells. Biochim. Biophys. Acta.

[25] El Kurdi R, Patra D (2017). The role of OH- in the formation of highly selective gold nanowires at extreme pH: multi-fold enhancement in the rate of the catalytic reduction reaction by gold nanowires. Phys. Chem. Chem. Phys..

[26] Al-Marhaby F, Seoudi R (2016). Preparation and characterization of silver nanoparticles and their use in catalytic reduction of 4-Nitrophenol. World J. Nano Sci. Engineer..

