# Supplementary material for: Solid-State Green Synthesis of Ag NPs: Higher Temperature Harvests Larger Ag NPs but Smaller Size Has Better Catalytic Reduction Reaction
Source: Sci Rep. 2019 Oct 23;9:15212. doi: 10.1038/s41598-019-51693-w (PMC6811623; doi:10.1038/s41598-019-51693-w)
Supplement: Supplementary file 1 — Supplemental Material [file 41598_2019_51693_MOESM1_ESM.pdf]

Supporting Information:-

Solid-State Green Synthesis of Ag NPs: Higher Temperature Harvests Larger Ag NPs but Smaller Size Has Better Catalytic Reduction Reaction

*Dina Sadeq Al-Namil, Elsy El Khoury, Digambara Patra**
Department of Chemistry, American University of Beirut, Beirut, Lebanon
Email: dp03@aub.edu.lb

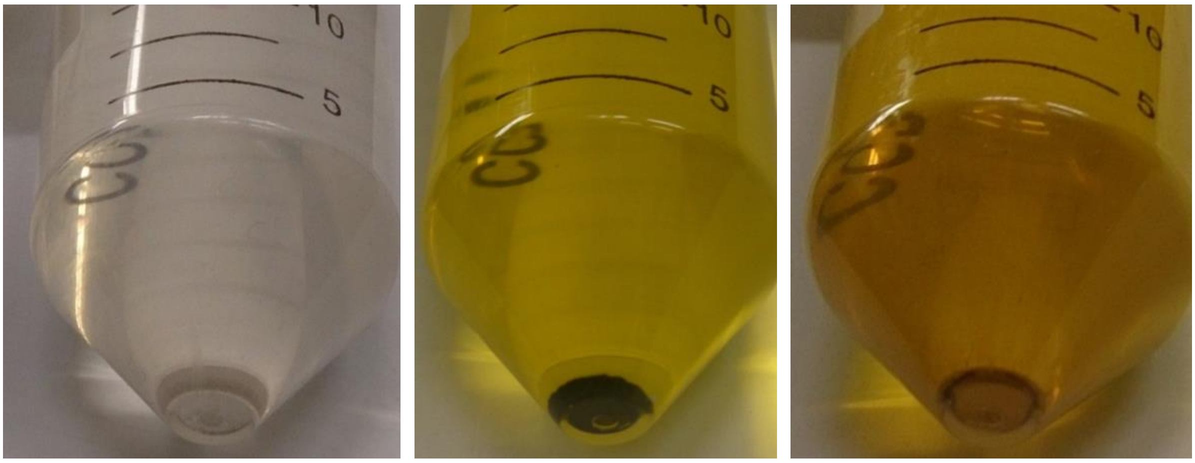

A: Control **B: Sample 1** **C: Sample 2**

Figure S1: Control, Sample 1 and Sample 2 after one cycle of centrifugation.

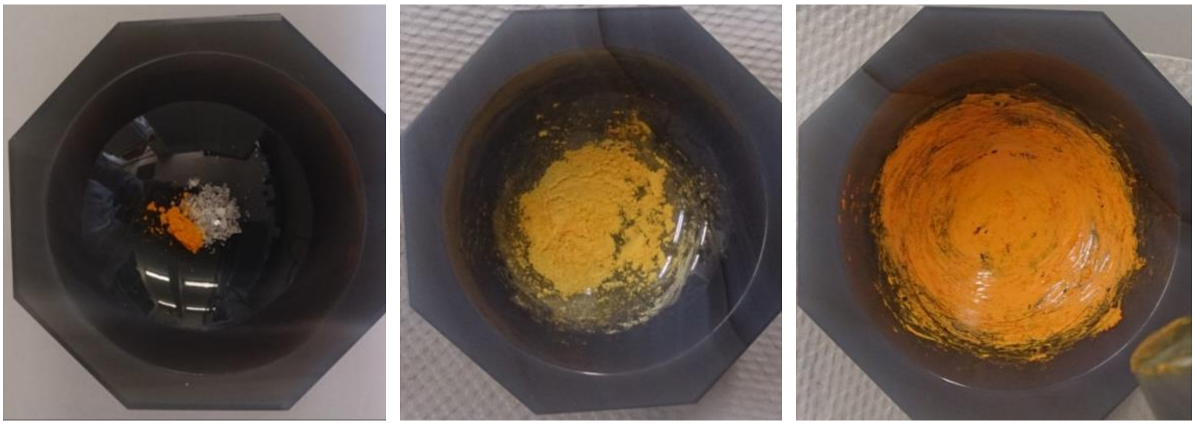

1 mmol of $AgNO_3$ + 0.025 mmol curcumin before mixing | **1 mmol of $AgNO_3$ + 0.05 mmol curcumin after gentle mixing before grinding** | **1 mmol $AgNO_3$ + 0.05 mmol curcumin after grinding for 10 minutes**

Figure S2: Change of color of curcumin during solid state synthesis of curcumin in the presence of $AgNO_3$ after grinding for 10 minutes in a marble mortar using a pestle.

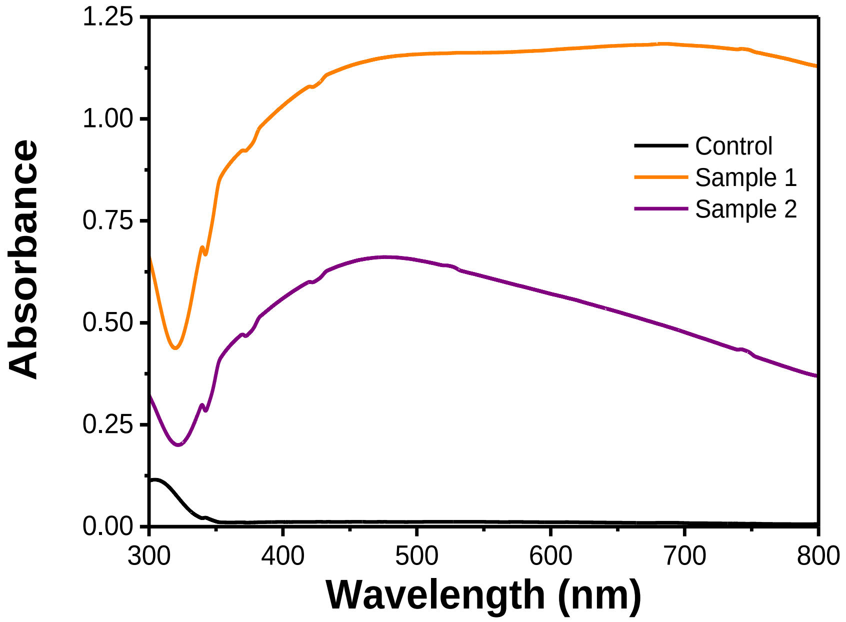

Figure S3: UV-Vis absorption spectra of control and Samples 1 and 2 after centrifugation and washing.

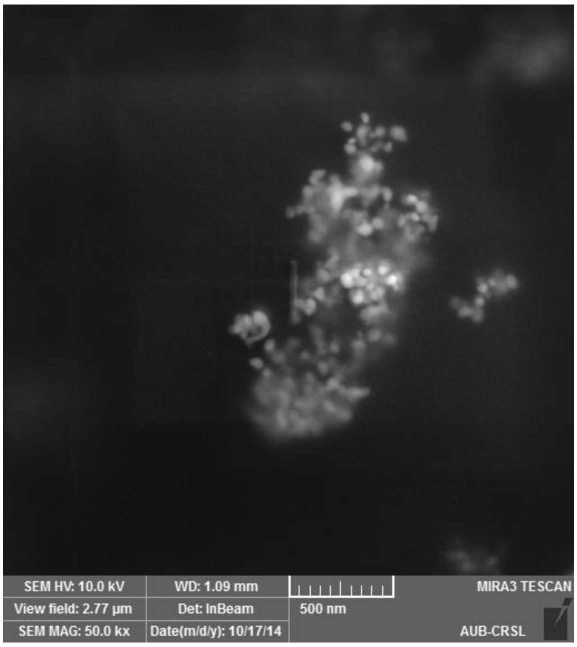

EDX results

Element	C	O	Ag
AgNPs 25 μmol curcumin (wt%)	49	4	47
AgNPs 50 μmol curcumin (wt%)	44	5	51

Figure S4: SEM image of Ag NPs prepared by solid state synthesis using curcumin (Sample 2) and EDX results.

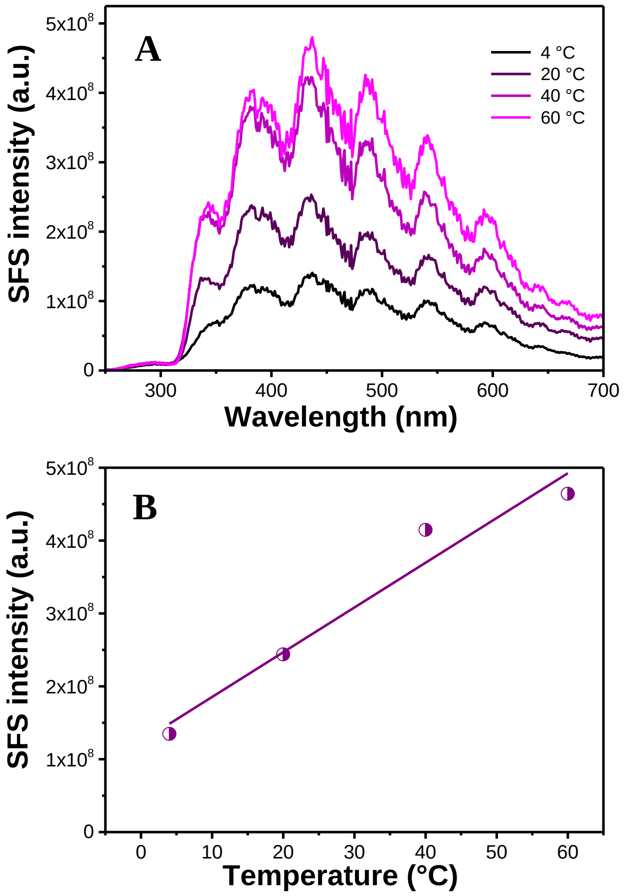

Figure S5: (A) Synchronous fluorescence spectra of curcumin mediated Ag NPs prepared by green solid state procedure at different temperatures after 1 day; (B) Variation of synchronous fluorescence (SFS) intensity with temperature during preparation of curcumin mediated Ag NPs prepared by green solid state procedure after 1 day.

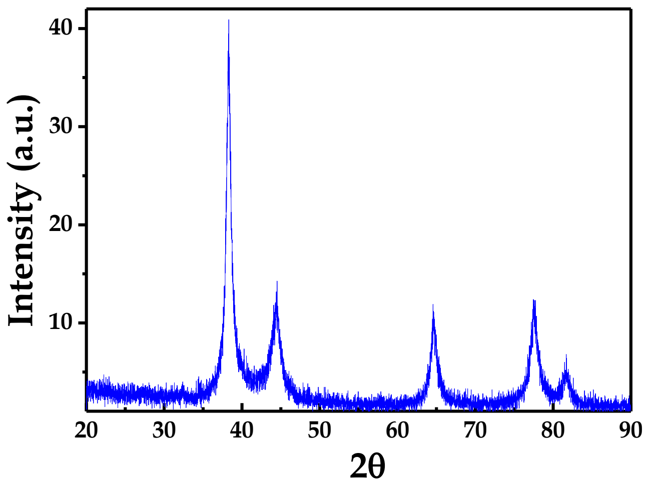

Figure S6: XRD pattern of curcumin conjugated Ag NPs prepared after 1day at 60°C;

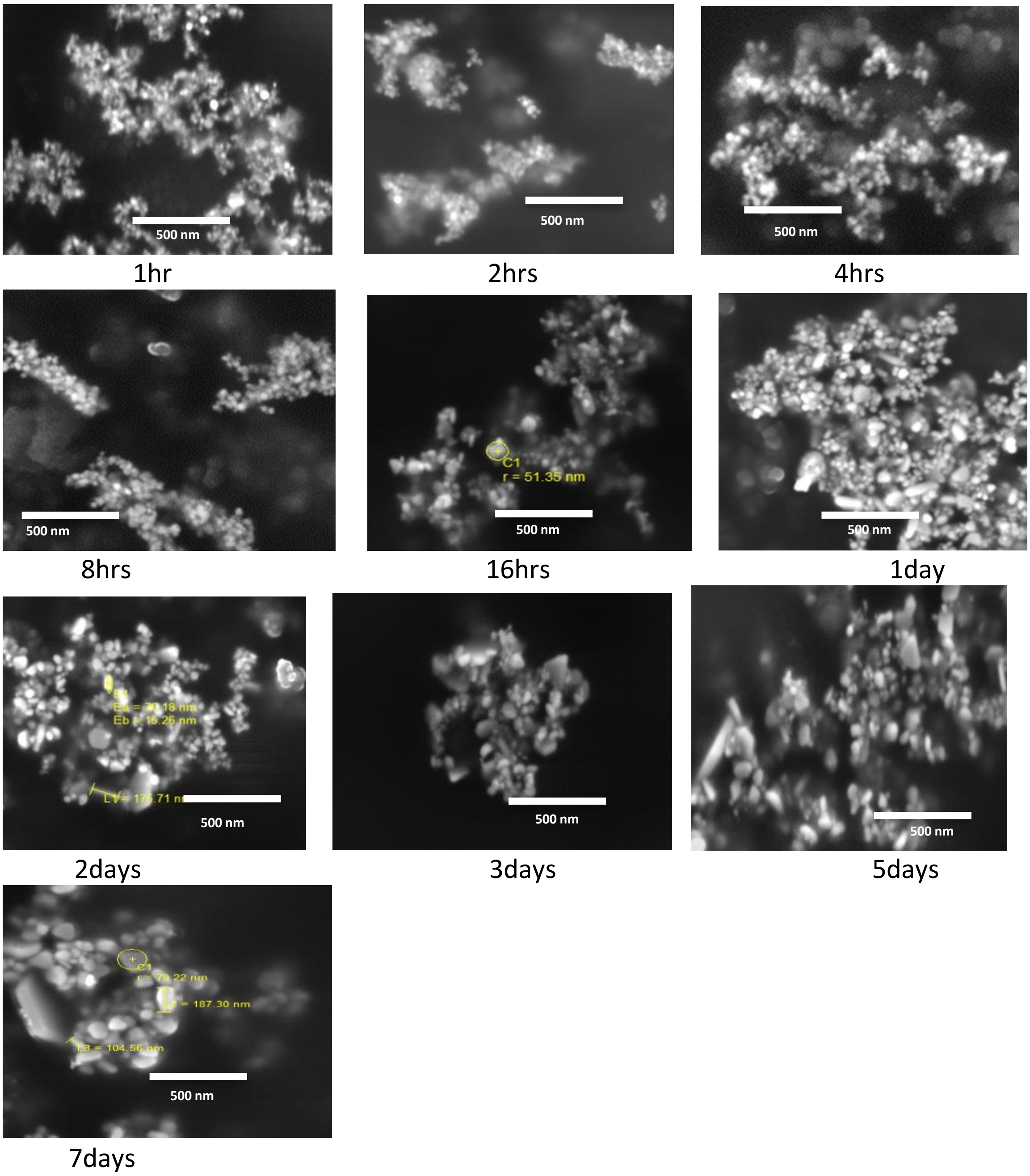

Figure S7: SEM images of curcumin conjugated Ag NPs in different growth time intervals at 20˚C.

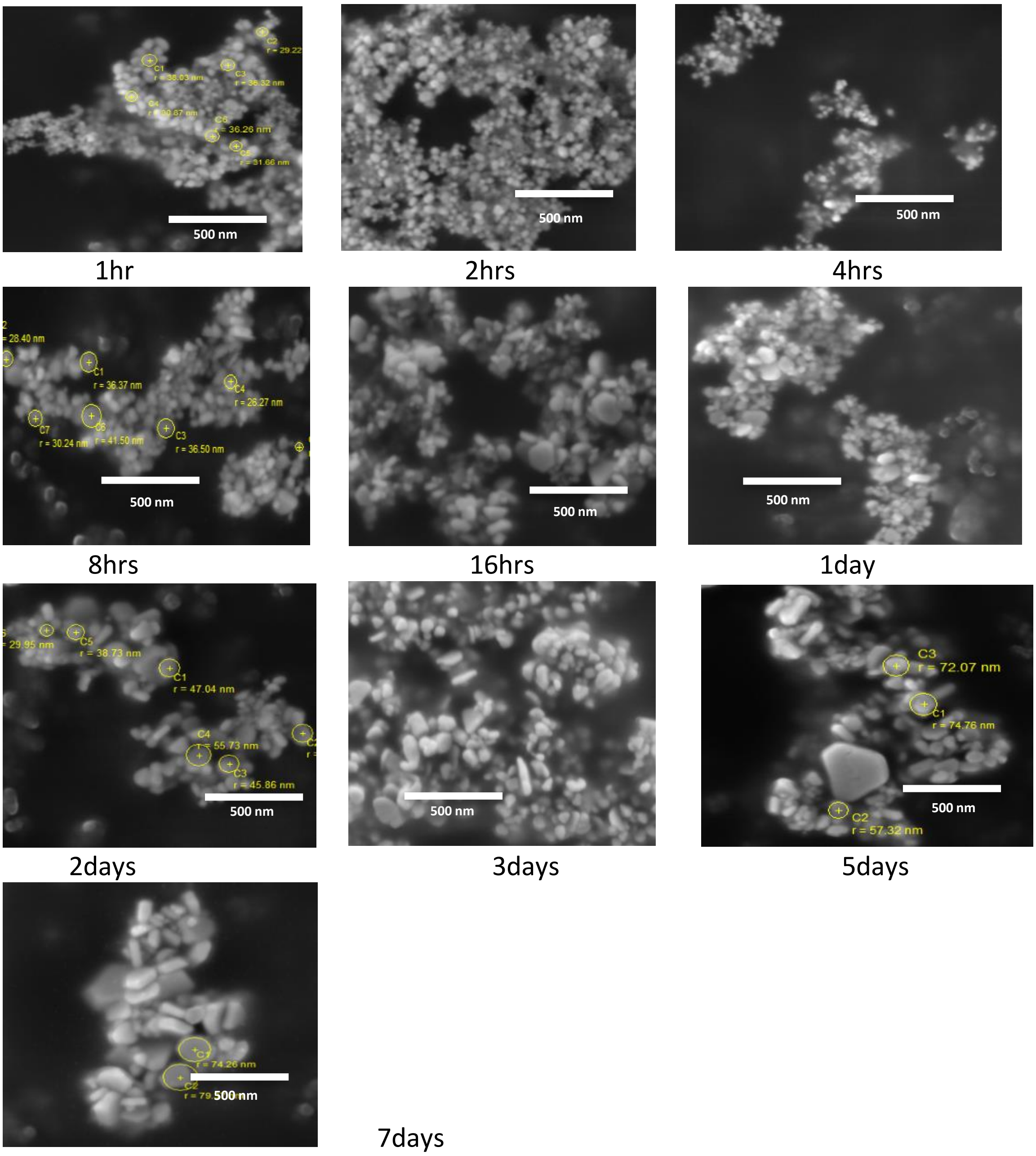

Figure S8: SEM images of curcumin conjugated Ag NPs in different growth time intervals at 40˚C.

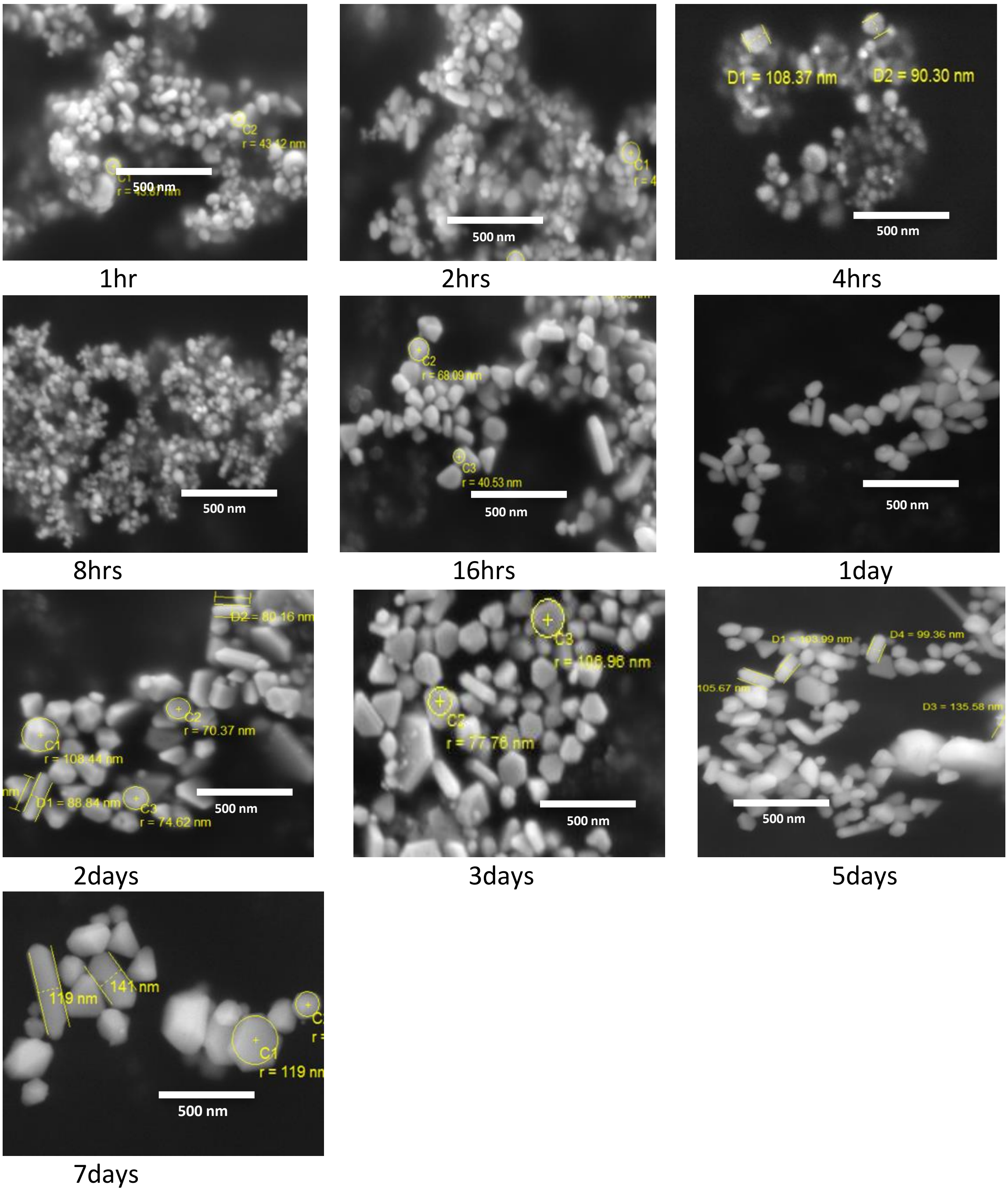

Figure S9: SEM images of curcumin conjugated Ag NPs in different growth time intervals at 60˚C.
